# Understanding exercise addiction, psychiatric characteristics and use of anabolic androgenic steroids among recreational athletes – An online survey study

**DOI:** 10.3389/fspor.2022.903777

**Published:** 2022-08-01

**Authors:** Björn Gunnarsson, Artin Entezarjou, Fernando Fernández-Aranda, Susana Jiménez-Murcia, Göran Kenttä, Anders Håkansson

**Affiliations:** ^1^Helsingborg University Hospital, Region Skåne, Helsingborg, Sweden; ^2^Department of Clinical Sciences in Malmö, Center for Primary Health Care Research, Lund University, Malmö, Sweden; ^3^Skåne University Hospital, Region Skåne, Malmö, Sweden; ^4^Department of Psychiatry, University Hospital of Bellvitge, Barcelona, Spain; ^5^CIBER Fisiopatología Obesidad y Nutrición (CIBERObn), Instituto de Salud Carlos III, Madrid, Spain; ^6^Department of Clinical Sciences, School of Medicine and Health Sciences, University of Barcelona, Barcelona, Spain; ^7^Psychoneurobiology of Eating and Addictive Behaviors Group, Neurosciences Programme, Bellvitge Biomedical Research Institute (IDIBELL), Barcelona, Spain; ^8^The Swedish School of Sport and Health Sciences, Stockholm, Sweden; ^9^School of Human Kinetics, University of Ottawa, Ottawa, ON, Canada; ^10^Swedish Sport Federation, Stockholm, Sweden; ^11^Clinical Sports and Mental Health Unit, Malmö Addiction Center, Region Skåne, Malmö, Sweden; ^12^Division of Psychiatry, Department of Clinical Sciences Lund, Faculty of Medicine, Lund University, Lund, Sweden

**Keywords:** exercise dependence, anabolic androgenic steroids, behavioral addiction, sports psychology, mental health

## Abstract

**Background:**

The purpose of this paper was to explore maladaptive behaviors among physically active individuals, including exercise dependence and use of anabolic steroids. Both exercise addiction (EA) and use of anabolic androgenic steroids (AAS) correlate to high amounts of exercise and EA have been linked to eating disorders and other mental health problems.

**Methods:**

An internet survey was spread through fitness-related social media. Inclusion criteria were age ≥ 15 years and exercise frequency ≥ thrice weekly. Exercise addiction inventory identified those at-risk of EA (rEA). Characteristics of rEA were compared to those not at risk. In a separate analysis, AAS users were compared to AAS-naïve individuals.

**Results:**

In total, 3,029 participants completed the questionnaire. Of these, 11% screened positive for being rEA, and 23% for ED. Factors associated with EA included daily exercise, social phobia, eating disorders and OCD. Risk consumption of alcohol was a negative predictor. Thirty seven participants had taken AAS the last year. These were mainly men, bodybuilders/powerlifters and more often used amphetamines and opioids.

**Discussion:**

This exploratory study supports EA being strongly associated with eating disorders. Identified associations between EA and compulsive or anxiety disorders warrant further research to clarify if these associations arise prior to, together with, or secondary to EA.

## Introduction

Exercise has shown many positive effects (Bouchard et al., 1994; McAuley and Rudolph, 1995), including cardiovascular benefits (Zimmer and Bloch, 2015; Wewege et al., 2018) and efficacy in treatment of several mental health disorders (Zschucke et al., 2013; Gordon et al., 2018). However, already in the 1970s negative effects from compulsive exercise were examined, whereas exercise addiction (EA) was first clearly described in a case study by Griffiths (Griffiths, 2009). It is suggested that EA is based on six criteria; salience, mood modification, tolerance, withdrawal, conflicts and relapse. It is described as a condition where the exercising individual is acting in a compulsive manner and exhibits dependence which leads to maladaptive effects for the individual – both socially and mentally (Griffiths et al., 2005).

The prevalence of EA in the general population is still disputed, with inconsistent findings in research. There appears to be a sport-specific variation in prevalence, with endurance sports ranging from 2.7 to 20% (Hausenblas and Downs, 2002; Youngman and Simpson, 2014; Zeulner et al., 2016; Mayolas-Pi et al., 2017). In more typical fitness populations symptoms of EA have been reported in the range of 6.8 to 10.2% (Lichtenstein et al., 2017; Rudolph, 2017). Moreover, sports such as CrossFit (5.0%) (Lichtenstein and Jensen, 2016) and soccer (7.1%) (Lichtenstein et al., 2014) have also been examined. Importantly, existing studies on exercising populations must critically discuss the ability to estimate the prevalence of EA in the general population, given that 31% of the world population does not meet the physical activity recommendations (Kohl et al., 2012). One study has, however, suggested an EA-prevalence of 3.6% in the “general exercising population” (Szabo and Griffiths, 2007). When looking at a general population in Hungary, it was found that between 0.3–0.5% of the general population were at risk of EA (Mónok et al., 2012).

EA has been linked to several other psychiatric diagnoses. It is believed that eating disorders (ED) such as anorexia nervosa or bulimia nervosa could lead to secondary EA in the form of compulsive exercise (Veale, 1987; Dalle Grave et al., 2008; Zeulner et al., 2016; Levallius et al., 2017). In these cases, ED should be treated together with EA, rather than separately, as the ED is most likely the major concern and the secondary EA is a sign of more severe ED (Strober et al., 1997; Solenberger, 2001; Dalle Grave et al., 2008; Levallius et al., 2017; Young et al., 2018). Combined ED and EA has previously been studied (Veale, 1987; Strober et al., 1997; Solenberger, 2001; Dalle Grave et al., 2008; Zeulner et al., 2016; Rudolph, 2017; Young et al., 2018) and there is an obvious correlation, although it is unclear whether EA always is secondary to ED. Muscle dysmorphia (MD), an assumingly underdiagnosed and more common disease, could potentially show signs of ED but is rather secondary to the exercise (Phillips et al., 2010; Giardino and Procidano, 2012; Tod et al., 2016; Klimek et al., 2018). Previous studies show that among weightlifters, 13–44% have a lifetime prevalence of MD (dos Santos Filho et al., 2015) and they rarely identify with the disorder (Olivardia, 2001).

Other addictive behaviors, such as the use of anabolic androgenic steroids (AAS) are also more common among recreational athletes than the general population (Parkinson and Evans, 2006) and associated with higher exercise frequency and training load (Ip et al., 2011). However, these performance-enhancing drugs have negative consequences, such as higher risk of tendon injury (Kanayama et al., 2015; Lindqvist Bagge et al., 2017), depression (Pope and Katz, 1994; Kicman and Gower, 2003; Ip et al., 2012; Lindqvist Bagge et al., 2017) and anxiety (Ip et al., 2011, 2012; Lindqvist Bagge et al., 2017), all with a dose-response-dependent increase in risk (Lindqvist Bagge et al., 2017). Liver dysfunction is common when AAS is orally administered (Kicman and Gower, 2003). Prolonged use is believed to be associated with risk of cardiovascular events (Sullivan et al., 1998; Pärssinen et al., 2000; Kicman and Gower, 2003; Hartgens et al., 2004; Bonetti et al., 2008; Baggish et al., 2017; Rasmussen et al., 2018), hypogonadism and infertility (Bonetti et al., 2008; Christou et al., 2017). It is however disputed whether these effects are reversible (Kicman and Gower, 2003; Christou et al., 2017; Rasmussen et al., 2018), but the psychiatric consequences are evident (Pärssinen et al., 2000; Ip et al., 2011, 2012; Piacentino et al., 2015).

Historically, only substance-related disorders have been recognized as addictive disorders by the international diagnostic systems (DSM-5, ICD-10). However, since DSM-5, the first non-substance use disorder is recognized as an addictive disorder – gambling addiction (Grant and Chamberlain, 2016). This calls for further investigation on behavioral addictions, such as EA, to possibly determine and recognize other behavioral addictions as mental disorders. The main purpose of this study is to examine EA and AAS-use among regular exercisers by using a cross-sectional survey with a large cohort. More specifically, it will be examined whether EA co-occurs with other addictive or obsessive conditions or not by comparing the characteristics of those at-risk of EA compared to other regular exercisers who are not at risk of EA. As a secondary aim, the prevalence and characteristics of active AAS users are compared to AAS-naïve regular exercisers.

## Methods

This study was conducted with ethical approval from the regional ethics board, Lund, Sweden (file number 2017/822).

### Participants

The survey addressed individuals in the general population but addressed those responding that they exercised regularly, at least three times weekly, and who were above the age of 15, and the survey opened only if the individual provided informed consent. Participants were recruited from April 1 through April 10, 2018, by spreading the survey through social media pages of several major Swedish health- and exercise-related blogs. Data was collected anonymously by Patient Information Broker (PIB) and I-Mind, Sweden.

### Materials

The survey consisted of several screening tools and questions were translated into Swedish. The exercise addiction inventory (EAI) is a six-question based screening tool for EA where the participant chooses from 1–5 on a Likert scale where 1 = “strongly disagree”, 5 = “strongly agree” (total scores between 6 and 30). This tool was chosen as it is not sport-specific, and easy to use. The cut-off level used for the screening of being at-risk if EA has been reported to be a score of 24 and above (at-risk of EA) (Griffiths et al., 2005).

The SCOFF (Sick, Control, One stone, Fat, Food) was used for the screening for ED, and consists of five yes-or-no questions screening for ED. Two or more affirmative answers indicate a likely ED. The test has both a high specificity and sensitivity (Morgan et al., 1999).

The AUDIT alcohol consumption questions (AUDIT-C) is a short 3-question screening version of the Alcohol Use Disorders Identification Test (AUDIT) and only focuses on consumption. Two items result in 0–4 points per question (total score between 0 and 12). Women with three or more points, or men with four or more points, are considered to have a hazardous use of alcohol (Bush et al., 1998).

Besides the screening tools listed above, the survey contained questions multiple choice-questions about exercise type (body-builder, runner, other endurance training, other) and exercise frequency (days per week, 3–7). Yes/no questions regarding a variety of psychiatric disorders, smoker status and past-year use of illicit drugs or AAS were given with the third option “would not like to respond”. Two questions were used to screen for problematic gambling, this was used for a separate study regarding problematic gambling among athletes. Demographic information included questions about gender (male/female/transgender) and age (categorized into age categories: 15–18, 19–24, 25–29, 30–39, 40–49, 50–59, and 60+ years). A categorical age distribution was chosen in order to facilitate the reporting by subjects on an online screen format, and age categories choses were the same as used in previous online surveys distributed through the same instrument (for example by Broman and co-workers in 2018 (Broman and Håkansson, 2018).

### Procedure

The participants accessed the study survey online through a link on social media. Before starting the questionnaire, the participants had to read the terms and consent to them. After the survey was finished, participants were given the results regarding EAI and if they felt they had a problematic relationship to exercise they were encouraged to contact a health professional.

### Statistical analyses

IBM SPSS 24.0.0.1 was used to analyse all data.

Participants were divided into two groups based on EAI score: those not at risk of EA (score of 6–23) and at-risk of EA (scores >23). The characteristics of these groups were then compared with each other using the Pearson chi-square test. Factors significantly associated with being at risk of EA (*p* < 0.05) were forwarded to a binary logistic egression analysis. Due to the relatively elevated number of factors controlled for, a conservative cut-off was set for significant levels in the logistic regression analysis, applying a Bonferroni correction.

Those who answered they had used AAS were separated and compared to those who reported being AAS-naïve using Fisher's exact test (due to the lower number of respondents endorsing this problem behavior). Due to the limited sample size in this analysis, only factors with *p* < 0.001 were inserted in a regression analysis, and a Bonferroni correction was applied in the interpretation of the logistic regression.

## Results

In Table 1, the characteristics of all participants are shown. Out of the 3,419 who started the survey, 3,029 (89%) completed all the questions. The majority of participants were between 25–39 years of age (54.4%), of male gender (63.8%), working (80.4%) and reported that their primary type of exercise was bodybuilding/powerlifting (58.7%). The mean EAI-score was 18.06 (SD ± 4.347). Among participants, 344 (11.0%) had an EAI-score >23 and qualified as at-risk. Table 1 displays the binary comparisons made between subjects being at-risk of EA compared to subjects not at risk of EA. After moving variables with significant associations to a binary logistic regression analysis, Table 2 shows the results of the regression analysis of potential correlates of being at risk of EA. The significant risk-factors were daily exercise and a high SCOFF-score. None of the substance use variables were related to being at risk of EA after the Bonferroni correction, although a high consumption of alcohol was negatively associated with being at-risk of EA in the uncorrected analysis. Having felt a need for psychiatric treatment, and being diagnosed with an eating disorder, social phobia, or OCD, were associated with being at risk of EA in the uncorrected analysis, but lost their significant associations after the Bonferroni correction.

**Table 1:** Descriptive characteristics of 3,029 athletes who exercise more than three times a week comparing those at risk of exercise addiction (EA) to those not at risk based on the exercise addiction inventory >23.

	**All subjects (*N =* 3,029)**	**Missing data, n**	**At risk of EA, *n* (%), *n =* 344**	**Not at risk of EA, *n* (%), *n =* 2685**	***p*-value**
Male gender	1,932 (64)	0	191 (56)	1741 (65)	0.001
Age groups Age 15–18 Age 19–24 Age 25–29 Age 30–39 Age 40–49 Age 50–59 Age 60+	48 (2) 393 (13) 657 (22) 996 (33) 681 (22) 222 (7) 321 (11)	0	10 (3) 57 (17) 74 (22) 113 (33) 69 (20) 21 (6) 0 (0)	38 (1) 336 (13) 583 (22) 883 (33) 612 (23) 201 (7) 32 (1)	0.002
Alcohol risk consumption	1,824 (60)	0	188 (55)	1,636 (61)	0.025
THC use	229 (8)	12	22 (6)	207 (8)	0.391
Benzodiazepine use	49 (2)	6	11 (3)	38 (1)	0.014
Cocaine use	78 (3)	10	13 (4)	65 (2)	0.135
Amphetamine use	47 (2)	8	8 (2)	39 (1)	0.217
Prescription opioid use	188 (6)	7	25 (7)	163 (6)	0.393
Taken ADHD medication without valid prescription	35 (1)	5	6 (2)	29 (1)	0.280
AAS use	37 (1)	7	8 (2)	29 (1)	0.047
Primarily runners	397 (13)	0	47 (14)	351 (13)	0.760
Primarily bodybuilders	1,778 (59)	0	202 (59)	1,576 (59)	0.993
Daily exercise	615 (20)	0	104 (30)	511 (19)	<0.001
Felt the need for psychiatric assessment	1,157 (38)	18	182 (53)	975 (37)	<0.001
Been in contact with a psychiatric health professional	912 (30)	11	132 (38)	780 (29)	<0.001
Lie/Bet positive	233 (8)	0	22 (6)	211 (8)	0.338
SCOFF-score > 1	694 (23)	0	159 (46)	535 (20)	<0.001
**Ever diagnosed with:**		
Eating disorder	118 (4)	7	36 (10)	82 (3)	<0.001
Panic disorder	188 (6)	6	37 (11)	151 (6)	<0.001
Social phobia	75 (2)	6	21 (6)	54 (2)	<0.001
General anxiety disorder	193 (6)	8	33 (10)	160 (6)	0.010
OCD	28 (1)	7	9 (3)	19 (1)	<0.001
ADD/ADHD	62 (2)	9	10 (3)	52 (2)	0.235
Depression	477 (16)	13	81 (24)	396 (15)	<0.001

**Table 2:** Binary logistic regression of factors significantly associated with being at-risk of exercise addiction compared to participants not at risk of exercise addiction.

	***p*-value**	**Adjusted *P*-value**	**OR (CI 95%)**	**Lower**	**Upper**
Male gender	0.654	^***^	0.941	0.722	1.227
Age groups	0.111	^***^	1.085	0.982	1.199
Alcohol risk-consumption	0.020	^***^	0.751	0.590	0.955
Benzodiazepine-usage	0.218	^***^	1.606	0.756	3.409
AAS-usage	0.150	^***^	1.885	0.796	4.464
Daily exercise	<0.001	<0.05	1.886	1.449	2.455
Felt the need for psychiatric assesment	0.003	^***^	1.635	1.178	2.269
Been in contact with a psychiatric health professional	0.117	^***^	0.734	0.499	1.080
SCOFF-score > 1	<0.001	<0.05	2.875	2.232	3.704
**Ever diagnosed with:**			
Eating disorder	0.007	^***^	1.933	1.198	3.118
Panic-disorder	0.489	^***^	1.185	0.733	1.915
Social phobia	0.015	^***^	2.185	1.167	4.092
General anxiety disorder	0.255	^***^	0.744	0.447	1.238
OCD	0.019	^***^	2.821	1.182	6.729
Depression	0.509	^***^	1.138	0.775	1.671

*** *Indicates non-significant values after Bonferroni correction*.

### AAS use

Out of the 3,029 participants, 37 responded they had been taking AAS the past year and seven chose not to respond. In Table 3 the characteristics of those using AAS are shown, compared to those who responded as AAS-naïve, the seven non-responders excluded. Table 4 displays the results of a regression analysis of the factors with a *p*-value < 0.001, showing a substantial correlation to bodybuilding and male gender as well as a positive correlation to taking prescription opioids and amphetamine, but not other substances.

**Table 3:** Descriptive characteristics of 3,022 athletes who exercise more than three times a week comparing those who reported using anabolic androgenic steroids (AAS-use) to those reporting not using anabolic androgenic steroids the last year, p-value after using Fisher's exact test.

	**AAS-use, n (%), *n =* 37**	**Not using AAS, n (%), *n =* 2,985**	**p-value**
Male gender	35 (94)	1891 (63)	<0.001
Age groups Age 15–18 Age 19–24 Age 25–29 Age 30–39 Age 40–49 Age 50–59 Age 60+	0 (0) 3 (8) 14 (38) 11 (30) 8 (22) 0 (0) 1 (3)	48 (2) 390 (13) 639 (21) 984 (33) 672 (23) 221 (7) 31 (1)	0.538
Alcohol risk consumption	22 (60)	1,796 (60)	0.528
THC use	9 (24)	219 (7)	0.001
Benzoiazepine use	3 (8)	46 (2)	0.021
Cocaine use	5 (14)	73 (2.4)	0.002
Amphetamine use	6 (16)	41 (1)	<0.001
Prescription opioid use	9 (24)	179 (6)	<0.001
Taken ADHD medication without valid prescription	4 (11)	31 (1)	0.001
Primarily runners	0 (0)	398 (13)	0.005
Primarily bodybuilders	36 (97)	1,735 (58)	<0.001
Daily exercise	9 (24)	605 (20)	0.331
Felt the need for psychiatric assessment	14 (38)	1,140 (38)	0.545
Been in contact with a psychiatric health professional	11 (30)	900 (30)	0.554
Lie/Bet positive	4 (11)	229 (8)	0.318
SCOFF-score > 1	13 (35)	679 (23)	0.061
Risk of Exercise Addiction	8	335	0.051
**Ever diagnosed with:**		
Eating disorder	0 (0)	118 (4)	0.226
Panic disorder	3 (8)	185 (6)	0.408
Social phobia	1 (3)	74 (2)	0.608
General anxiety disorder	5 (14)	188 (6)	0.084
OCD	0 (0)	28 (1)	0.707
ADD/ADHD	3 (8)	59 (2)	0.039
Depression	9 (24)	467 (18)	0.118

**Table 4:** Binary logistic regression of factors associated with having used anabolic androgenic steroids the last year compared to participants reporting not using anabolic androgenic steroids the last year.

	***p*-value**	**Adjusted *P*-value**	**OR (CI 95%)**	**Lower**	**Upper**
Male gender	0.004	0.016	8.255	1.968	34.628
Primarily bodybuilders	0.003	0.012	20.635	2.814	151.304
Amphetamine use	<0.001	0.004	6.687	2.340	19.112
Prescription opioid use	0.006	0.024	3.342	1.412	7.912

### Eating disorder

There were 118 participants who reported being diagnosed with ED; their mean EAI-score was 21.36 (SD ± 4.404). Of those at risk of EA there were 36 (10.7%) diagnosed with an ED, 33 female and 3 males. A *post-hoc* analysis of those who said bodybuilding/powerlifting was their primary form of exercise showed a significantly increased risk of having a SCOFF-score>1 (*p* < 0.005), compared to those who said they primarily participated in running, endurance sports or other sports.

## Discussion

The main result of this study is that 11% of a physically active self-selected population are at-risk of EA. In the same group, AAS use was primarily used by bodybuilders/powerlifters. A prevalence of 11% at-risk of EA is quite high, but still within the estimates of previous studies (Hausenblas and Downs, 2002; Lichtenstein et al., 2014, 2017; Youngman and Simpson, 2014; Lichtenstein and Jensen, 2016; Zeulner et al., 2016; Mayolas-Pi et al., 2017; Rudolph, 2017). What might have influenced, giving the somewhat higher prevalence could be the fact that this study used a minimum of exercising days as inclusion, especially since daily exercise was associated to EA and increasing amount of exercise has previously been shown to be associated with EA (Lichtenstein and Jensen, 2016; Lichtenstein et al., 2017; Rocks et al., 2017). Even though this study did not specify the amount in hours like previous studies, the same correlation can be seen in amount of days exercised. It can, however, not be excluded that some individuals exercised more than once on exercise days.

In our results, no correlation could be found between at-risk of EA and depression, while a Danish study found that not only did those at-risk of EA more frequently show symptoms of depression compared to the general population, but also that the risk of depression was significantly increased in case of injury (Lichtenstein et al., 2018). This explorative study specifically asked for diagnosed mental disorders, while the Danish one used the self-report questionnaire HADS. It is believed that many use exercise as a form of relief of emotional distress (i.e., reducing anxiety), and might not have contacted any medical expertise. Thus, it is possible that undiagnosed depression is more prevalent even in our study population. Exercise withdrawal has shown to lead to mood-disturbances among regular exercisers (Chan and Grossman, 1988; Szabo, 1995; Mondin et al., 1996), and in case of a dependence the risk of suffering from psychological distress could be worse. Moreover, the finding that those at-risk of EA felt the need to seek out a mental health professional – but did not – could be argued to further support this notion.

Among the mental disorders asked for, social phobia and OCD had equally strong correlation as ED to EA. A previous study found that anxiety of body appearance often was associated to EA (Cook et al., 2015), but to the authors knowledge, social anxiety disorder is a variable never studied before, and should be further investigated. OCD and ED, being compulsive disorders which correlate to EA, could open for a discussion about whether EA might rather be characterized as a compulsive disorder rather than an addictive disorder.

In this study population 23% had a positive SCOFF-test, which is relatively high, but in agreement with previous studies among athletes. Up to 45% of female and up to 19% of male athletes showed symptoms of ED (Bratland-Sanda and Sundgot-Borgen, 2013), whilst among the general population the lifetime prevalence is 1% (Jie et al., 2013). Because of the difficulty to differentiate between secondary EA and ED due to exercise, this study only focused on EA rather than dividing them into separate groups. However, the majority of participants were bodybuilders/powerlifters, a group more susceptible to MD (Phillips et al., 2010; Skemp et al., 2013) which has been shown to involve restricted eating (Murray et al., 2010; Giardino and Procidano, 2012; Devrim et al., 2018). Both MD and ED are associated with EA (Giardino and Procidano, 2012), and it is clear in the results that this group should be further investigated regarding ED as only 36 were diagnosed, whilst 164 had a SCOFF-score > 1 without diagnosis.

Previous data in both animal and human studies has suggested exercise to reduce drug seeking behavior on a neurological level (Robertson et al., 2016; Robison et al., 2018). In our sample a negative correlation between high alcohol consumption and being at-risk of EA was found, which could further support this claim. Participants taking AAS were however more frequently using other substances, which is in agreement with previous studies (Ip et al., 2011, 2012; Kersey et al., 2012; Mhillaj et al., 2015; McDuff et al., 2019) and it has been shown that the effects of stimulants is decreased and the drug-seeking behavior in general is increased (Mhillaj et al., 2015). This group of individuals should most likely be examined selectively because of the comorbidity they may develop based on the AAS-use alone.

This study has several limitations. Firstly, the self-report of diagnoses referred to a history of receiving an actual diagnosis, which requires the participant to have sought medical attention, compared to using self-report questionnaires where even undiagnosed disorders can be found. This could influence the results of mental disorders in this study. Being a retrospective survey, nothing can be said about the causality, only the correlation can be examined.

In conclusion, looking at the results from this study, many respondents at-risk of EA also had one or more psychiatric diagnoses. A causality could not be determined in this study, but the risk of depression in case of withdrawal is known since before (Lichtenstein et al., 2018). Screening and assessing those with EA for other mental illnesses could enable treatment and help them achieve a healthier relationship to exercise, and the risk of adverse reactions in case of withdrawal should lessen. Those using AAS should be separately investigated, as their use of other substances makes it more complex and they are probably in need of different assessments than those at-risk of EA. Being at-risk of EA is particularly strongly associated with being at-risk of ED, while associations to compulsive or anxiety components warrants further prospective research to clarify if these associations arise prior to, together with, or secondary to EA.

## Data availability statement

The raw data supporting the conclusions of this article will be made available by the authors, without undue reservation.

## Ethics statement

The studies involving human participants were reviewed and approved by Regionala etikprövningsnämnden Lund/Regional Ethics Board Lund, Sweden. Written informed consent from the participants' legal guardian/next of kin was not required to participate in this study in accordance with the national legislation and the institutional requirements.

## Author contributions

BG, AE, FF-A, SJ-M, and AH planned the study. AH was responsible of the ethics application. AH, BG, and AE were part of the data collection procedure. BG conducted the statistical analyses, wrote the draft of the paper, and which was edited by all authors. GK added interpretations regarding sports psychology. FF-A and SJ-M added interpretations of clinical behavioral addiction aspects. Results were discussed by all authors. All authors contributed to the article and approved the submitted version.

## Funding

The study was finance using the general research funding of AH, from the regional health care organization and from the state-owned gambling operator AB Svenska Spel.

## Conflict of interest

Author AH has overall research funding from the state-owned gambling operator Svenska Spel, which however was not involved in the present study, which did not received any project-specific funding. Authors FF-A and SJ-M received consultancy honoraria from Novo Nordisk and editorial honoraria as EIC from Wiley (FF-A), which had no role in the study. The remaining authors declare that the research was conducted in the absence of any commercial or financial relationships that could be construed as a potential conflict of interest.

## Publisher's note

All claims expressed in this article are solely those of the authors and do not necessarily represent those of their affiliated organizations, or those of the publisher, the editors and the reviewers. Any product that may be evaluated in this article, or claim that may be made by its manufacturer, is not guaranteed or endorsed by the publisher.
